# T-Cell Large Granular Lymphocytic Leukemia: A First Case Report Diagnosed by Flow Cytometry in Vietnam

**DOI:** 10.7759/cureus.20249

**Published:** 2021-12-07

**Authors:** Do T Vinh An, Le Lan Anh, Nguyen Tuan Tung

**Affiliations:** 1 Hematology and Blood Transfusion, Bach Mai Hospital, Hanoi, VNM

**Keywords:** leukemia, large granular lymphocytes, t-cell immunophenotype, lymphocytic infiltration, lymphoproliferative

## Abstract

T-cell large granular lymphocytic leukemia (T-LGL leukemia) is a rare, chronic lymphoproliferative disorder in the peripheral blood. This is characterized by peripheral blood and bone marrow (BM) lymphocytic infiltration with clonal large granular lymphocytes (LGLs). The neoplastic cells of this disease display a mature T-cell immunophenotype, with the majority of cases showing a CD4-/CD8+ T-cell, T-cell receptor (TCR) subset immunophenotype versus other permutations of those markers.

## Introduction

T-cell large granular lymphocytic leukemia (T-LGL leukemia) is a rare chronic mature lymphoproliferative neoplasia (for more than six months) [1,2]. This disease is characterized by lymphocytic infiltration in the peripheral blood and marrow with clonal LGLs, involving tissue invasion of the skin, spleen, and liver. Lymphadenopathy is very rare [1]. T-LGL leukemias show a constitutive mature post-thymic phenotype, which was immunocytochemically positive for CD3, CD8, and CD2. CD57 and CD16 expression are strong in more than 80% of the population. Some rare subsets of LGL leukemia may have CD4+ with or without co-expression of CD8, T-cell receptor (TCR) gamma delta, CD5, and CD7 [1,3,4]. The standard treatment of LGL leukemia is immunosuppressive therapies using low-dose methotrexate (with or without prednisolone combination) or other drugs such as cyclosporine and cyclophosphamide. Alemtuzumab is being studied for the treatment of persistent cases. Calcitriol (the active form of vitamin D) is partially effective according to some statistical data [5-7]. Here, we report the first case of T-LGL leukemia newly diagnosed in Vietnam by flow cytometric analysis.

## Case presentation

A 49-year-old female patient was admitted with complaints of fever, abdominal bloating, and losing weight for one year. She was diagnosed with cirrhosis and was treated at a local hospital. Three months ago, the patient deteriorated; thus, peripheral blood test, bone marrow (BM) aspiration, and bone marrow biopsy were performed. The results showed lymphocytosis in the marrow. Therefore, the patient was referred to our center. On physical examination, she had a fever (about 38°C), mild pallor, swollen legs, mild hepatomegaly, and huge splenomegaly. There was no purpura and no peripheral lymphadenopathy. There were no clinical infections and no joint damage.

**Figure 1:**
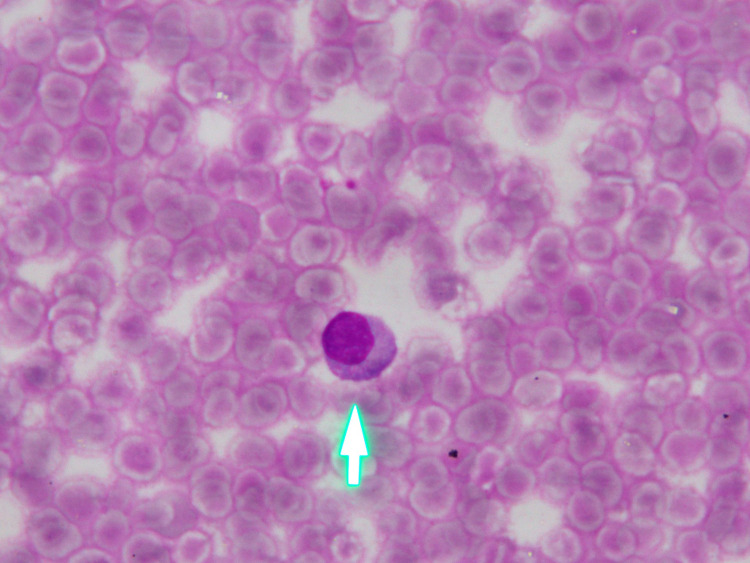
Peripheral blood smear The lymphocytes were predominantly large lymphocytes, which were having abundant cytoplasm containing coarse azurophilic granules and clumped chromatin (arrow)

Complete hemogram revealed hemoglobin of 117 g/L, platelet count of 82 G/L, total leukocyte count of 12.63 G/L with 65% lymphocytes, and 30% neutrophils. The peripheral blood smear showed lymphocytosis and thrombocytopenia (Figure 1). The lymphocytes were predominantly large lymphocytes, which were having abundant cytoplasm containing coarse azurophilic granules and clumped chromatin. Her biochemical examination was fairly normal. Serological examination revealed no evidence of HIV, HBV, HCV, EBV, CMV, or dengue infection. The results of cultures of fungi and bacteria in blood were negative. The ultrasound of the abdomen confirmed mild hepatomegaly and huge splenomegaly.

**Figure 2:**
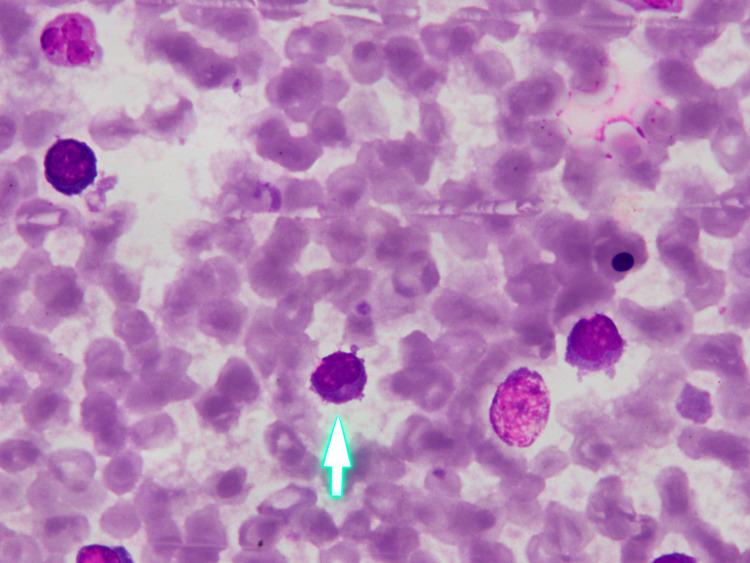
Bone marrow aspiration BM showing large granular lymphocytes (lymphocytosis) (arrow)

**Figure 3:**
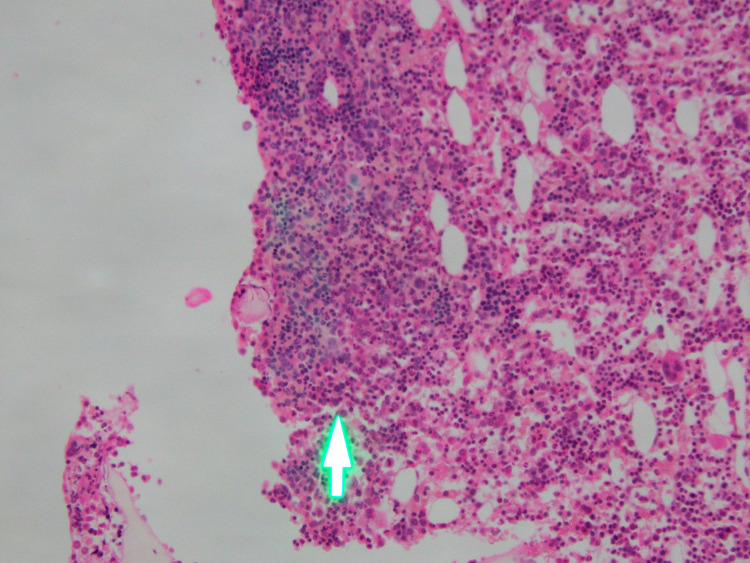
Bone marrow biopsy BM increasing level of cell density, lymphocytic infiltration in hematopoietic compartments (arrow)

Bone marrow imprint smears showed 33% lymphocytes (lymphocytosis) (Figure 2). The lymphocytes displayed a medium to large size with a moderate amount of cytoplasm containing numerous azurophilic granules and a round nucleus with clumped chromatin. Bone marrow biopsy displayed an increasing level of cell density and lymphocytic infiltration in hematopoietic compartments with nonuniform size and similar morphology lymphocytes seen in peripheral smear (Figure 3). The erythroid, myeloid, and megakaryocytic series were suppressed.

Cytogenetics revealed a normal karyotype. Flow cytometric analysis of the bone marrow showed that 49.5% of cells were of lymphoid origin. These lymphoid cells were positive for T-cell markers, including CD2, CD8, CyCD3, CD5, CD7, CD56, CD16, and TCR gamma delta. The cells expressed CD4-. These findings were suggestive of T-cell large granular lymphocytic leukemia.

After being treated with methotrexate for one month, the clinical condition was ameliorated. The patient had no fever and splenomegaly regression, and the peripheral blood revealed an increasing proportion of neutrophils (65%). Therefore, the patient was given outpatient treatment and kept being examined monthly.

## Discussion

T-cell large granular lymphocytic leukemia (T-LGL leukemia) is a rare chronic mature lymphoproliferative neoplasia (for more than six months), with the LGL count ranging from 2 to 20 G/L in the peripheral blood [1,2]. This disease is more prevalent among adults, mainly between 45 and 75 years old (73%) [8,9]. T-LGL leukemia demonstrates a strong association with autoimmune diseases, especially rheumatoid arthritis [9].

The patient in our report was a 49-year-old female with clinical presentations of fever, abdominal bloating, and losing weight. She was diagnosed with and being treated for cirrhosis for about one year. Unfortunately, the treatment was ineffective. During treatment, the patient was found to have lymphocytosis and was referred to our center. Here, further investigations were performed. Serology tests, such as HIV, HBV, HCV, CMV, EBV, and dengue, were negative. The results of cultures of fungi and bacteria in blood were negative. Thus, microorganism causes were possibly eliminated.

The peripheral blood in T-LGL leukemia reveals an increasing number of LGL count (>2 G/L), cytopenia (most commonly neutropenia), and anemia, with no significant change in the platelet count [1]. A lower number of LGL counts combining other relevant criteria may be compatible with the diagnosis [3,4,10]. Morphologically, the lymphocytes display a large size and an abundant cytoplasm containing typical azurophilic granules with mature chromatin. In the bone marrow, approximately >50% of cases are hyperplasia of lymphocytes. Granular lymphocytosis may be seen in the spleen [11]. The peripheral blood smear of this patient showed lymphocytosis; >50% of the lymphocytes contained azurophilic granules in the protoplasm (Figure 1). In the bone marrow, the proportion of lymphocytes was 33% (Figure 2), most of which had large size and granular cytoplasm. Bone marrow biopsy displayed lymphocytosis in the hematopoietic compartments (Figure 3).

When determining the immunophenotype of the cells by flow cytometry, the result showed that most lymphocyte populations were mature T-cells with CD2, CD5, and CD7 expression. The CD8 marker was very bright in this lymphoid population (>70%), and it was simultaneously positive with markers CD56 and CD16. The TCR gamma delta marker was also strongly positive in this upper lymphoid population. According to 2016 WHO diagnostic standards, this is a typical immunophenotype of large granular T-cell leukemia [12], with the first-line treatment being methotrexate (with or without prednisolone combination) [5-7].

## Conclusions

T-cell large granular lymphocytic leukemia (T-LGL leukemia) is one of the rare chronic mature lymphoproliferative disorders (about 2%-3% of all cases). The diagnosis of LGL leukemia can be a dilemma without cell morphology and full panel immunophenotyping.

## References

[1] Jaffe ES, Harris NL, Stein H, Vardiman JW (2001). Pathology and genetics of tumours of haematopoietic and lymphoid tissues. World Health Organization classification of tumours.

[2] Kwong YL, Wong KF (1998). Association of pure red cell aplasia with T large granular lymphocyte leukaemia. J Clin Pathol.

[3] Chan WC, Link S, Mawle A, Check I, Brynes RK, Winton EF (1986). Heterogeneity of large granular lymphocyte proliferations: delineation of two major subtypes. Blood.

[4] Pandolfi F, Loughran TP Jr, Starkebaum G (1990). Clinical course and prognosis of the lymphoproliferative disease of granular lymphocytes. A multicenter study. Cancer.

[5] Sanikommu SR, Clemente MJ, Chomczynski P (2018). Clinical features and treatment outcomes in large granular lymphocytic leukemia (LGLL). Leuk Lymphoma.

[6] Rosenblum MD, LaBelle JL, Chang CC, Margolis DA, Schauer DW, Vesole DH (2004). Efficacy of alemtuzumab treatment for refractory T-cell large granular lymphocytic leukemia. Blood.

[7] Olson KC, Kulling PM, Olson TL, Tan SF, Rainbow RJ, Feith DJ, Loughran TP Jr (2017). Vitamin D decreases STAT phosphorylation and inflammatory cytokine output in T-LGL leukemia. Cancer Biol Ther.

[8] Shah MV, Hook CC, Call TG, Go RS (2016). A population-based study of large granular lymphocyte leukemia. Blood Cancer J.

[9] Bareau B, Rey J, Hamidou M (2010). Analysis of a French cohort of patients with large granular lymphocyte leukemia: a report on 229 cases. Haematologica.

[10] Lamy T, Loughran TP (1998). Large granular lymphocyte leukemia. Cancer Control.

[11] Lamy T, Loughran TP Jr (2003). Clinical features of large granular lymphocyte leukemia. Semin Hematol.

[12] Kojić Katović S, Vasilj A, Rinčić G (2018). T-cell large granular lymphocytic leukemia - case reports. Acta Clin Croat.

